# Healthy Eating Index-2020 and bowel habits: a cross-sectional analysis of NHANES

**DOI:** 10.3389/fnut.2025.1578124

**Published:** 2025-06-13

**Authors:** Rongpeng Chen, Zexin Fu, Zhicheng Feng, Feng Xiao, Guoqiang Wang

**Affiliations:** ^1^Department of Gastrointestinal Surgery, The Second Affiliated Hospital of Guangzhou Medical University, Guangzhou, China; ^2^Department of Ultrasound, The Eighth Affiliated Hospital of Sun Yat-sen University, Shenzhen, China

**Keywords:** HEI-2020, fecal incontinence, diarrhea, constipation, bowel habits

## Abstract

**Background:**

Dietary habits play crucial roles in gastrointestinal health. The relationship between dietary patterns, measured using the Healthy Eating Index-2020 (HEI-2020), and bowel habits remains unclear. This study aimed to explore the associations between HEI-2020 scores and bowel habits, including fecal incontinence, diarrhea, and constipation, in adults in the US.

**Methods:**

This cross-sectional study included 11,590 participants of the National Health and Nutrition Examination Survey. Multivariate logistic regression models were used to assess the associations adjusted for demographic, socioeconomic, and health-related covariates. Weighted quantile sum (WQS) regression was used to evaluate the combined effects of the dietary components.

**Results:**

Analysis of 11,590 individuals showed that higher HEI-2020 scores were negatively correlated with fecal incontinence and constipation. When treated as a continuous variable, HEI-2020 scores were associated with reduced odds of fecal incontinence (weighted adjusted OR: 0.86, 95% CI: 0.79–0.95, *p* = 0.004) and constipation (weighted adjusted OR: 0.78, 95% CI: 0.73–0.84, *p* < 0.001). In quartile analysis, the highest HEI-2020 quartile was linked to a 48% lower constipation risk compared with the lowest quartile (weighted adjusted OR: 0.52, 95% CI: 0.40–0.68, *p* < 0.001). Subgroup analysis indicated that higher HEI-2020 scores were more strongly associated with a reduced constipation risk in participants with sleep disorders. WQS regression revealed significant protective effects of HEI-2020 scores on fecal incontinence and constipation, but not on diarrhea.

**Conclusion:**

Higher HEI-2020 scores were associated with a reduced risk of fecal incontinence and constipation. Adherence to the HEI-2020 guidelines may enhance gastrointestinal health by mitigating abnormalities in bowel habits.

## 1 Introduction

Gastrointestinal health, particularly bowel habits, is a critical indicator of overall health and quality of life. In contemporary society, a dietary pattern characterized by the widespread consumption of highly processed foods, added sugars, and saturated fats has emerged. This dietary shift coincides with a rising incidence of functional gastrointestinal disorders, such as constipation, diarrhea, and fecal incontinence (1, 2). These conditions not only impose significant physical discomfort, but also contribute substantial economic burdens on healthcare systems and societies worldwide, highlighting the urgent need to identify modifiable dietary factors.

Dietary patterns play a pivotal role in modulating gastrointestinal function and bowel habits. A high-quality diet can promote gut motility, maintain intestinal microbiota homeostasis, and prevent various bowel disorders, whereas poor diet may disrupt these physiological processes and increase the risk of bowel habit abnormalities (3, 4). While individual nutrients such as fiber are known to affect stool bulk and transit time (5), emerging evidence suggests that the combined effect of dietary components may be more influential than isolated interventions (6–8). However, most epidemiological studies have focused on single nutrients or specific clinical cohorts, overlooking the role of overall dietary quality in shaping bowel function across diverse populations.

The Healthy Eating Index-2020 (HEI-2020), a measure of diet quality based on the Dietary Guidelines for Americans, comprehensively evaluated dietary patterns across 13 components including adequacy and moderation. Higher HEI-2020 scores indicate better adherence to the recommended dietary guidelines, which emphasize the consumption of fruits, vegetables, whole grains, dairy, and protein while limiting the intake of added sugars, saturated fats, and sodium (9). Previous studies have documented associations between HEI scores and various health outcomes, such as cardiovascular diseases, diabetes, and certain cancers (10–12). However, few studies have explored the relationship between the HEI-2020 scores and bowel habits in a nationally representative sample of adults.

To address this, this study investigated the association between HEI-2020 scores and bowel habits in a nationally representative adult population using data from the National Health and Nutrition Examination Survey (NHANES) 2005–2010. The findings of this study may provide valuable insights into the role of dietary quality in preventing and managing bowel habit disorders, and may inform public health strategies and dietary recommendations for improving gastrointestinal health in the general adult population.

## 2 Materials and methods

### 2.1 Study population in NHANES

This cross-sectional study utilized data from three 2-year cycles of the NHANES, spanning the period from 2005 to 2010. The data used in this analysis are publicly accessible through the NHANES database, which is a comprehensive health and nutritional status survey that includes demographic, dietary, screening, laboratory, and questionnaire data. All study protocols were approved by the Ethics Review Board of the National Center for Health Statistics and written informed consent was obtained from all participants prior to data collection. Detailed NHANES study design and data are publicly available at www.cdc.gov/nchs/nhanes/. This study adhered to the Strengthening the Reporting of Observational Studies in Epidemiology (STROBE) reporting guidelines (13).

**Figure 1:**
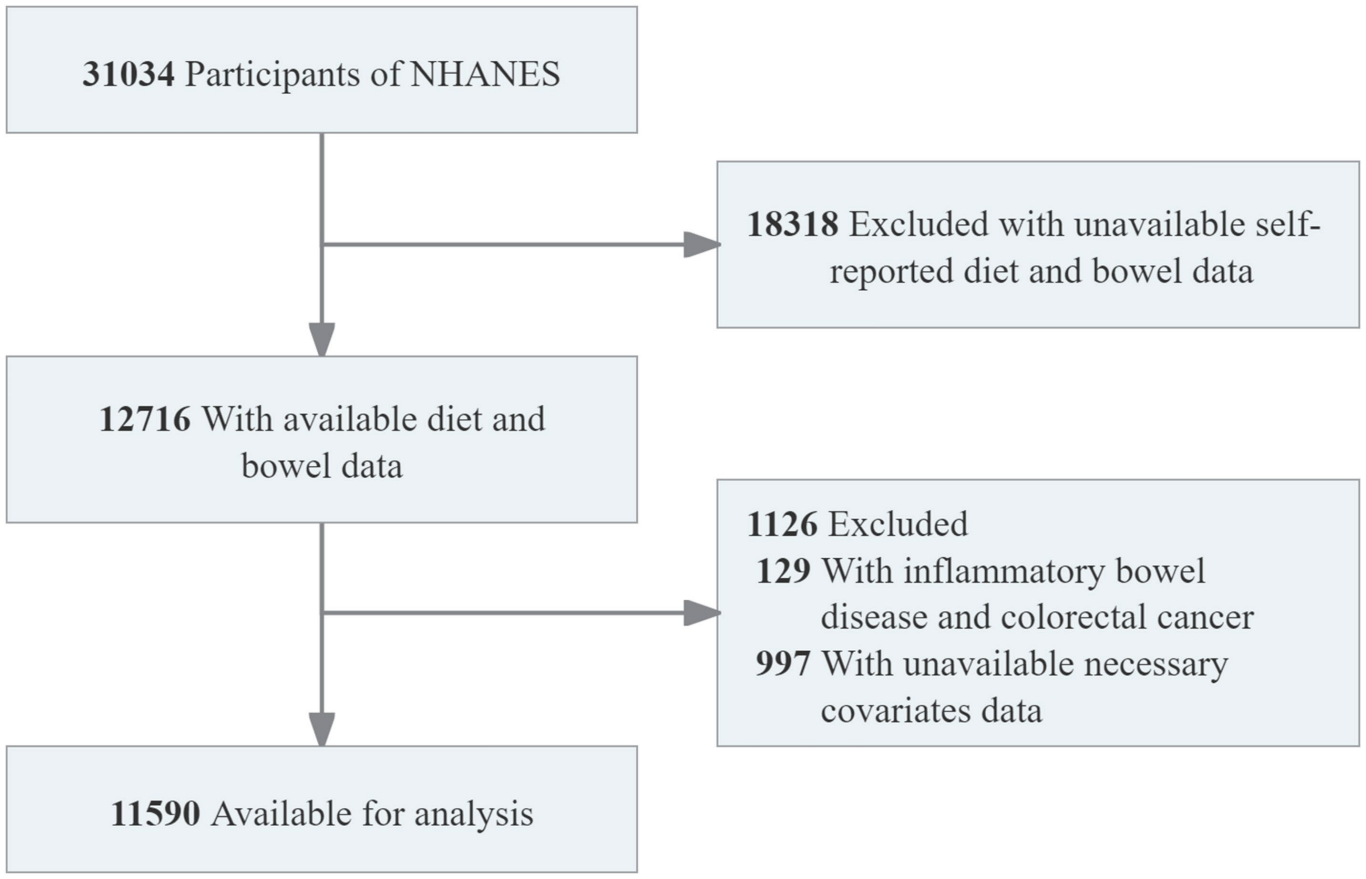
Flowchart of sample selection from NHANES 2005–2010.

The Bowel Health Questionnaire was administered to adults aged 20 years and older and was included in the NHANES database only from 2005 to 2010. Therefore, only the participants from this period were included in the analysis. The study initially included 31,034 participants. Individuals with missing data on the Bowel Health Questionnaire and HEI-2020 scores (n = 18,318) were excluded. Individuals with a history of inflammatory bowel disease or colorectal cancer (n = 129) were also excluded. Less than 5% of the covariate data related to demographics, lifestyle, and comorbidities were missing and were handled by deletion. The process of participant selection and exclusion is illustrated in Figure 1. Ultimately, 11,590 participants were included in the analysis, representing approximately 153.26 million adults in the United States.

### 2.2 HEI-2020 score

The HEI-2020 score was derived from 13 components, comprising nine adequacy and four moderation components, to evaluate adherence to the 2020–2025 Dietary Guidelines for Americans. The scores ranged from 0 (lowest adherence) to 100 (highest adherence) (9).

### 2.3 Dietary intake assessment

To comprehensively assess daily food intake, participants were asked to provide two 24-h dietary recalls covering factors such as total energy, protein, fat, sugar, and other nutrients. The initial interview was conducted face-to-face, and the follow-up interview was conducted by telephone 3–10 days later. The HEI-2020 score was calculated by determining the mean of the two recalls (DR1TOT and DR2TOT) to assess dietary quality.

### 2.4 Assessment of bowel habits

The Bowel Health Questionnaire, administered during the 2005–2010 NHANES, was used to assess the participants’ bowel habits and stool characteristics. The participants were first asked whether they had any unintentional bowel leakage, which included four types: gas leakage, mucus leakage, liquid stool leakage, and solid stool leakage. They were then asked how often they typically experienced bowel movements. Participants were then instructed: “Please look at this card and tell me the number that corresponds to your usual or most common stool type.” At the same time, they were shown a card depicting the seven types of the Bristol Stool Form Scale (BSFS; Types 1–7), each accompanied by a colored picture and description. Based on previous studies (14–17), fecal incontinence was defined as the involuntary loss of mucus, liquid, or solid stool at least once in the previous 30 days. Constipation was defined based on participants’ reported bowel movement frequency of ≤3 bowel movements per week or identification of their typical stool type as BSFS type 1 (separate hard lumps, like nuts) or type 2 (sausage-like but lumpy). Diarrhea was identified in participants who reported their typical stool type as BSFS type 6 (fluffy pieces with ragged edges, a mushy stool) or type 7 (watery, non-solid pieces).

### 2.5 Other covariates

The following covariates were selected for inclusion in the study based on existing literature and clinical relevance (14, 16, 18): demographic and socioeconomic factors (age, sex, race, education level, and family income-to-poverty ratio); lifestyle characteristics (body mass index (BMI), alcohol consumption, and physical activity); and comorbidities (diabetes, hypertension, and sleep disorders). Education level was categorized as “less than high school” and “high school or higher.” Income-to-poverty ratio was categorized into three groups: ≤ 1.30 (low), 1.31–3.50 (medium), and > 3.50 (high) to reflect household economic status. The BMI of the study population was calculated from height and weight measurements; BMI was expressed as weight divided by height squared (kg/m^2^). Alcohol consumption was defined as the consumption of at least 12 alcoholic drinks per year. Self-reported physical activity at work or during recreational activities was classified as vigorous, moderate, or neither vigorous nor moderate. Diabetes mellitus was identified through self-reported physician-diagnosed diabetes, use of oral hypoglycemic agents or insulin, or a hemoglobin A1c level ≥6.5%. Hypertension was defined as systolic/diastolic blood pressure ≥140/90 mmHg or the use of antihypertensive medications. Sleep disorders were identified by self-reporting of physician-diagnosed sleep disorders.

### 2.6 Statistical analysis

In accordance with the NHANES analytical guidelines, this study accounted for the complex survey design of the NHANES and sample weights from Mobile Examination Center tests. In the present study, HEI-2020 scores were considered both continuous and categorical variables, with the latter divided into quartiles. Continuous variables are presented as mean ± standard error (SE) or median (interquartile range) [Q2 (Q1, Q3)]. Categorical variables were presented as frequencies (percentages). Continuous variables were compared using the t-test, while categorical data were analyzed using the χ^2^ test.

In this study, we used multivariate logistic regression to examine the association between the HEI-2020 scores and bowel habits. Based on univariate logistic regression analyses, covariates were selected and incorporated into the multivariate model (Supplementary Table 1). Prior to analysis, the HEI-2020 scores were standardized using z-score transformation. For the sensitivity analysis, the HEI-2020 scores were analyzed both as continuous variables and quartiles. We constructed three models for logistic regression: an unweighted crude model, an unweighted adjusted model, and a weighted adjusted model. The unweighted crude model was not adjusted for covariates. The unweighted adjusted model was additionally adjusted for age, sex, education level, family income, BMI, alcohol use, physical activity, diabetes, hypertension, and sleep disorders. The weighted adjusted model further incorporated survey weights to account for the complex survey design, in addition to adjusting for the above covariates. The results of the multivariate logistic analysis are presented using odds ratios (ORs) and 95% confidence intervals (CIs).

Restricted cubic splines (RCS) were used to model nonlinear dose–response relationships, and likelihood ratio tests were applied to evaluate the model fit against linear assumptions. Building on these analyses, we evaluated subgroup and interaction analyses to gain a more comprehensive understanding of these associations. To assess the combined effects of the HEI-2020 components, a weighted quantile sum (WQS) regression was used to estimate the mixture effects, with component weights constrained to preserve directionality.

Statistical significance was defined as a *p* value less than 0.05. All analyses were conducted using the R survey package (version 4.1) and the gWQS package with 1,000 bootstrap iterations. Analyses were also performed using the Free Statistics Analysis Platform (version 2.0).

## 3 Results

### 3.1 Participant characteristics

Table 1 shows the baseline characteristics of the study participants stratified by bowel habits. The final analysis included 11,590 individuals from NHANES 2005 to 2010, representing approximately 153.26 million non-institutionalized adults in the US aged ≥20 years. The cohort was 48.1% male and 51.9% female, with a mean age of 49.4 ± 17.8 years. The mean HEI-2020 score for all participants was 51.1 ± 11.7. In total, 1,044 participants were diagnosed with fecal incontinence, 850 with diarrhea, and 1,195 with constipation.

**Table 1:** Characteristics of participants in the NHANES 2005–2010 cycles.

Characteristic		Fecal Incontinence		*p* value	Diarrhea		*p* value	Constipation		*p* value
	Overall	Non-patients	Patients		Non-patients	Patients		Non-patients	Patients	
	*n* = 11,590	*n* = 10,546	*n* = 1,044		*n* = 10,740	*n* = 850		*n* = 10,395	*n* = 1,195	
HEI-2020 Continuous	51.1 ± 11.7	51.1 ± 11.7	51.1 ± 11.3	0.957	51.2 ± 11.7	50.6 ± 11.6	0.165	51.4 ± 11.7	48.9 ± 11.2	< 0.001
HEI-2020 Category				0.073			0.051			< 0.001
Q1	2,898 (25.0)	2,661 (25.2)	237 (22.7)		2,682 (25)	216 (25.4)		2,525 (24.3)	373 (31.2)	
Q2	2,897 (25.0)	2,604 (24.7)	293 (28.1)		2,655 (24.7)	242 (28.5)		2,573 (24.8)	324 (27.1)	
Q3	2,897 (25.0)	2,637 (25)	260 (24.9)		2,708 (25.2)	189 (22.2)		2,624 (25.2)	273 (22.8)	
Q4	2,898 (25.0)	2,644 (25.1)	254 (24.3)		2,695 (25.1)	203 (23.9)		2,673 (25.7)	225 (18.8)	
Sex				0.003			< 0.001			< 0.001
Male	5,579 (48.1)	5,123 (48.6)	456 (43.7)		5,219 (48.6)	360 (42.4)		5,262 (50.6)	317 (26.5)	
Female	6,011 (51.9)	5,423 (51.4)	588 (56.3)		5,521 (51.4)	490 (57.6)		5,133 (49.4)	878 (73.5)	
Age	49.4 ± 17.8	48.5 ± 17.7	57.9 ± 16.3	< 0.001	49.1 ± 17.9	53.2 ± 16.6	< 0.001	49.7 ± 17.7	46.3 ± 18.3	< 0.001
Race				< 0.001			0.016			< 0.001
Mexican American	1997 (17.2)	1862 (17.7)	135 (12.9)		1830 (17)	167 (19.6)		1805 (17.4)	192 (16.1)	
Non-Hispanic	890 (7.7)	826 (7.8)	64 (6.1)		814 (7.6)	76 (8.9)		783 (7.5)	107 (9)	
White	6,008 (51.8)	5,394 (51.1)	614 (58.8)		5,615 (52.3)	393 (46.2)		5,460 (52.5)	548 (45.9)	
Black	2,250 (19.4)	2057 (19.5)	193 (18.5)		2069 (19.3)	181 (21.3)		1940 (18.7)	310 (25.9)	
Other	445 (3.8)	407 (3.9)	38 (3.6)		412 (3.8)	33 (3.9)		407 (3.9)	38 (3.2)	
Education				< 0.001			< 0.001			< 0.001
Less than high school	3,013 (26.0)	2,694 (25.5)	319 (30.6)		2,697 (25.1)	316 (37.2)		2,655 (25.5)	358 (30)	
High school or higher	8,577 (74.0)	7,852 (74.5)	725 (69.4)		8,043 (74.9)	534 (62.8)		7,740 (74.5)	837 (70)	
Family Income				< 0.001			< 0.001			< 0.001
Low	3,303 (28.5)	2,979 (28.2)	324 (31)		2,986 (27.8)	317 (37.3)		2,864 (27.6)	439 (36.7)	
Medium	4,456 (38.4)	4,019 (38.1)	437 (41.9)		4,144 (38.6)	312 (36.7)		3,985 (38.3)	471 (39.4)	
High	3,831 (33.1)	3,548 (33.6)	283 (27.1)		3,610 (33.6)	221 (26)		3,546 (34.1)	285 (23.8)	
Body Mass Index	29.1 ± 6.7	29.0 ± 6.7	30.2 ± 7.3	< 0.001	29.0 ± 6.6	30.7 ± 7.4	< 0.001	29.2 ± 6.7	28.4 ± 6.9	< 0.001
Alcohol Use				0.047			0.012			< 0.001
No	3,301 (28.5)	2,976 (28.2)	325 (31.1)		3,027 (28.2)	274 (32.2)		2,846 (27.4)	455 (38.1)	
Yes	8,289 (71.5)	7,570 (71.8)	719 (68.9)		7,713 (71.8)	576 (67.8)		7,549 (72.6)	740 (61.9)	
Physical Activity				< 0.001			< 0.001			< 0.001
Low	6,137 (53.0)	5,460 (51.8)	677 (64.8)		5,613 (52.3)	524 (61.6)		5,446 (52.4)	691 (57.8)	
Moderate	4,107 (35.4)	3,810 (36.1)	297 (28.4)		3,851 (35.9)	256 (30.1)		3,712 (35.7)	395 (33.1)	
High	1,346 (11.6)	1,276 (12.1)	70 (6.7)		1,276 (11.9)	70 (8.2)		1,237 (11.9)	109 (9.1)	
Diabetes				< 0.001			< 0.001			< 0.001
No	7,033 (60.7)	6,567 (62.3)	466 (44.6)		6,598 (61.4)	435 (51.2)		6,245 (60.1)	788 (65.9)	
Yes	4,557 (39.3)	3,979 (37.7)	578 (55.4)		4,142 (38.6)	415 (48.8)		4,150 (39.9)	407 (34.1)	
Hypertension				< 0.001			< 0.001			0.146
No	10,016 (86.4)	9,212 (87.4)	804 (77)		9,329 (86.9)	687 (80.8)		8,967 (86.3)	1,049 (87.8)	
Yes	1,574 (13.6)	1,334 (12.6)	240 (23)		1,411 (13.1)	163 (19.2)		1,428 (13.7)	146 (12.2)	
Sleep Disorders				< 0.001			< 0.001			0.047
No	8,814 (76.0)	8,148 (77.3)	666 (63.8)		8,236 (76.7)	578 (68)		7,933 (76.3)	881 (73.7)	
Yes	2,776 (24.0)	2,398 (22.7)	378 (36.2)		2,504 (23.3)	272 (32)		2,462 (23.7)	314 (26.3)	

### 3.2 Association between HEI-2020 scores and bowel habits

Table 2 presents the results of the three logistic regression models examining the association between the HEI-2020 scores and bowel habits. The analyses included unadjusted, unweighted adjusted, and weighted adjusted models. The multivariate regression analysis was adjusted for age, sex, education level, family income, BMI, alcohol use, physical activity, diabetes, hypertension, and sleep disorders. When HEI-2020 scores were treated as a continuous variable, a negative correlation was observed between HEI-2020 scores and fecal incontinence (weighted adjusted OR: 0.86, 95% CI: 0.79–0.95, *p* = 0.004) and constipation (weighted adjusted OR: 0.78, 95% CI: 0.73–0.84, *p* < 0.001), regardless of whether sampling weighting was applied. When HEI-2020 scores were categorized into quartiles, individuals in the highest quartile (Q4) had a 48% lower risk of constipation compared with those in the lowest quartile (Q1) (weighted adjusted OR: 0.52, 95% CI: 0.40–0.68, *p* < 0.001). For fecal incontinence, Q4 participants showed reduced odds in unweighted adjusted models (OR: 0.80, 95% CI: 0.66–0.97, *p* = 0.026), but this association lost statistical significance after weighting (OR: 0.77, 95% CI: 0.58–1.02, *p* = 0.066). Diarrhea exhibited no consistent trend across quartiles in the adjusted models, with only a modest protective effect observed in the weighted analyses.

**Table 2:** Multivariate logistic regression analysis of the association between HEI-2020 scores and bowel habits.

Variables	Unadjusted model		Unweighted adjusted model^a^		Weighted ^b^ adjusted model	
	OR (95%CI)	*p* value	OR (95%CI)	*p* value	OR (95%CI)	*p* value
Fecal Incontinence						
HEI-2020 Continuous	1.00 (0.94 ~ 1.06)	0.957	0.90 (0.84 ~ 0.96)	0.002	0.86 (0.79 ~ 0.95)	0.004
Category						
Q1	1 (Ref)		1 (Ref)		1 (Ref)	
Q2	1.26 (1.06 ~ 1.51)	0.011	1.12 (0.93 ~ 1.34)	0.240	1.11 (0.89 ~ 1.38)	0.347
Q3	1.11 (0.92 ~ 1.33)	0.279	0.94 (0.78 ~ 1.14)	0.548	0.93 (0.73 ~ 1.19)	0.575
Q4	1.08 (0.90 ~ 1.30)	0.423	0.80 (0.66 ~ 0.97)	0.026	0.77 (0.58 ~ 1.02)	0.066
Trend text		0.794		0.005		0.026
Diarrhea						
HEI-2020 Continuous	0.95 (0.89 ~ 1.02)	0.165	0.94 (0.87 ~ 1.02)	0.118	0.89 (0.81 ~ 0.99)	0.035
Category						
Q1	1 (Ref)		1(Ref)		1 (Ref)	
Q2	1.13 (0.93 ~ 1.37)	0.204	1.09 (0.90 ~ 1.33)	0.380	1.10 (0.86 ~ 1.41)	0.444
Q3	0.87 (0.71 ~ 1.06)	0.166	0.85 (0.69 ~ 1.05)	0.123	0.78 (0.60 ~ 1.02)	0.071
Q4	0.94 (0.77 ~ 1.14)	0.510	0.90 (0.73 ~ 1.10)	0.302	0.80 (0.61 ~ 1.05)	0.107
Trend text		0.143		0.081		0.024
Constipation						
HEI-2020 Continuous	0.81 (0.76 ~ 0.86)	<0.001	0.80 (0.75 ~ 0.86)	<0.001	0.78 (0.73 ~ 0.84)	<0.001
Category						
Q1	1 (Ref)		1 (Ref)		1 (Ref)	
Q2	0.85 (0.73 ~ 1.00)	0.049	0.84 (0.72 ~ 0.99)	0.040	0.94 (0.73 ~ 1.20)	0.595
Q3	0.70 (0.60 ~ 0.83)	<0.001	0.70 (0.59 ~ 0.83)	<0.001	0.76 (0.63 ~ 0.93)	0.010
Q4	0.57 (0.48 ~ 0.68)	<0.001	0.56 (0.46 ~ 0.67)	<0.001	0.52 (0.40 ~ 0.68)	<0.001
Trend text		<0.001		<0.001		<0.001

a Adjusted Model: Adjusted for age, sex, education level, family income, body mass index, alcohol use, physical activity, diabetes, hypertension, and sleep disorders.

b The analysis was conducted using data from the NHANES. Survey weights were applied to account for the complex survey design, including stratification, clustering, and unequal probability of selection. Estimates in the table are weighted to reflect the US population.

### 3.3 Dose–response relationship between HEI-2020 scores and bowel habits

**Figure 2:**
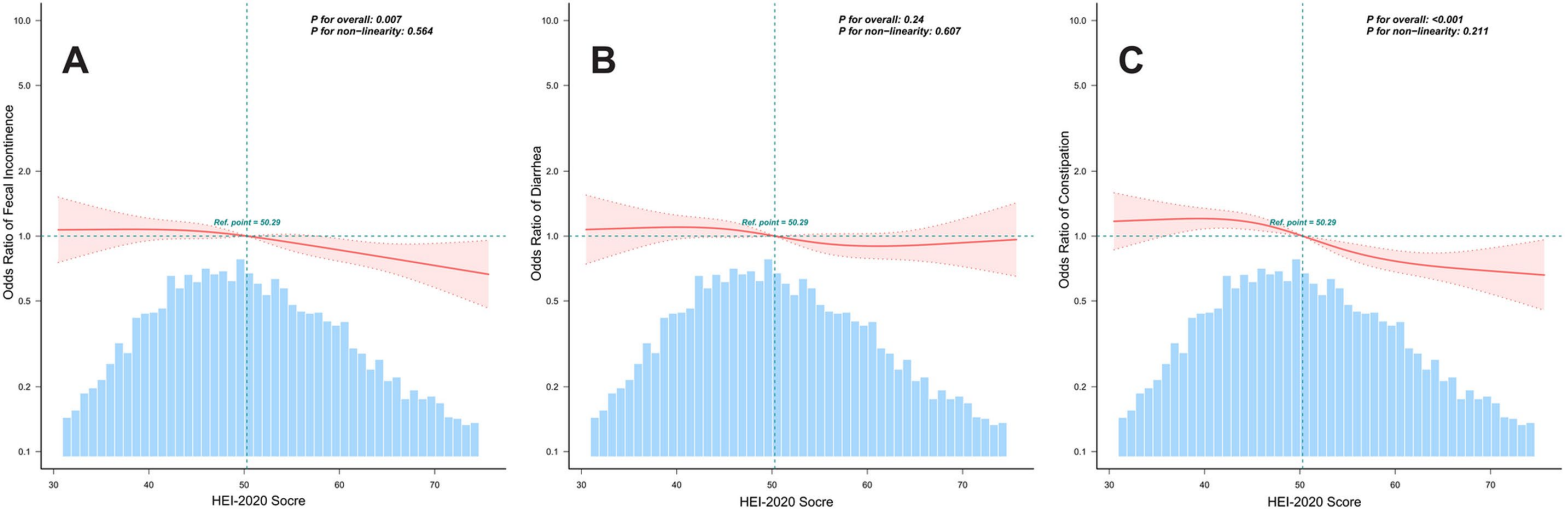
Analysis of restricted cubic splines regression. Dose–response relationships between HEI-2020 scores (continuous) and bowel habits, using RCS. Panels show the following models: **(A)** Fecal Incontinence Model. **(B)** Diarrhea Model. **(C)** Constipation Model. All models were adjusted for age, sex, education level, family income, body mass index, alcohol use, physical activity, diabetes, hypertension, and sleep disorders. Only 99% of the data is displayed.

We employed RCS to elucidate the dose–response relationship between HEI-2020 scores and bowel habits. As depicted in Figure 2, after adjusting for covariates, RCS analysis revealed an inverse dose–response relationship between the HEI-2020 scores and the risk of fecal incontinence (P for overall = 0.007) and constipation (P for overall < 0.001). Conversely, no significant dose–response relationship was observed between the HEI-2020 scores and the risk of diarrhea (P for overall = 0.240).

### 3.4 Subgroup analysis

Stratified analyses revealed that higher HEI-2020 scores were associated with a reduced risk of bowel habit disorders in nearly all the subgroups (Supplementary Table 2). Notably, stratified analyses based on the presence of sleep disorders showed that among participants with sleep disorders, higher HEI-2020 scores were more strongly associated with a reduced risk of constipation (OR: 0.71, 95% CI: 0.62–0.81, *p* < 0.001) compared with participants without sleep disorders (OR: 0.84, 95% CI: 0.78–0.80, *p* < 0.001), with a statistically significant interaction effect (P for interaction = 0.030). Supplementary Table 3 shows that further stratified analysis indicated that the combination of sleep disorders and a low HEI-2020 score increased the risk of constipation by 73.3% (OR = 1.733, 95% CI: 1.437–2.089, *p* < 0.001) and that 27.2% of the excess risk could be attributed to their synergistic effect (AP = 0.272, *p* = 0.002).

### 3.5 Mixed effects of 13 dietary components on bowel habits

We used the WQS regression model to assess the mixed effects of 13 distinct dietary components within the HEI-2020 scores on the three bowel habits. As shown in Supplementary Table 4, when the HEI-2020 score was treated as a mixture variable, a significant protective effect was observed for fecal incontinence (OR: 0.83, 95% CI: 0.72–0.97, *p* = 0.017) and constipation (OR: 0.82, 95% CI: 0.70–0.95, *p* = 0.008). However, no statistically significant association was found with diarrhea (*p* = 0.951).

We visualized the contributions of the 13 dietary components in the WQS regression model, as depicted in Supplementary Figure 1. For fecal incontinence, the fatty acid ratio (an adequacy component) contributed the most to the protective effect, followed by sodium intake (a moderation component), and whole fruit intake (an adequacy component), with weights of 0.3524, 0.2595, and 0.0878, respectively. For constipation, whole grain intake (an adequacy component) had the highest contribution, followed by sodium intake (a moderation component) and saturated fat intake (a moderation component), with weights of 0.3729, 0.1915, and 0.1846, respectively (Supplementary Table 5).

## 4 Discussion

The present study used nationally representative data from the NHANES to elucidate the association between adherence to the HEI-2020 dietary guidelines and bowel habit abnormalities.

Our findings revealed a robust, dose-dependent, inverse relationship between HEI-2020 scores and constipation risk. Individuals in the highest quartile exhibit nearly halved odds compared to the lowest quartile (weighted adjusted OR: 0.52, 95% CI: 0.40–0.68, *p* < 0.001). The WQS regression analysis identified whole grains (an adequacy component) as the dominant protective factor, accounting for 37.3% of the weight. Sodium intake (a moderation component) was the second most influential factor, contributing 19.2% of the weight.

This association was further supported by the findings of Rollet et al. in the ORISCAV-LUX 2 survey (19). Their analysis demonstrated that grains, lipid-rich foods, total fat, and starch intake were associated with lower constipation scores, whereas sugary products, sodium, and higher energy intake were associated with higher constipation in adults living in Luxembourg. However, when investigating the association between individual dietary components of the HEI-2020 score and constipation using multivariate logistic regression analysis, no significant association was observed between sodium intake and constipation risk (Supplementary Table 6). This suggests that the combined effects of dietary components may be more influential than the individual interventions.

The identification of whole grains as the primary contributing component in the WQS regression model suggests a potential mechanistic link between dietary fiber intake and the pathophysiology of constipation. Whole grains likely exert their effects through their dual roles as insoluble fiber sources and microbiota-modulating substrates (5, 20). Whole grains such as wheat, oats, rye, and rice are rich in indigestible dietary fiber. This fiber significantly increases stool weight and frequency. This effect is partly due to the water-absorbing and swelling properties of fiber, which increase stool volume and water content, thereby softening feces and improving bowel habits. Although different whole grains have slightly varying effects on the stool volume, all are superior to refined grains (21, 22). Additionally, components of whole grains, such as arabinoxylans and oligosaccharides, can act as prebiotics to promote the proliferation of beneficial bacteria, such as bifidobacteria and lactobacilli (23). Long-term consumption of whole grains, such as whole wheat breakfast cereals or wheat bran, can significantly increase the abundance of bifidobacteria in feces. This demonstrates a pronounced prebiotic effect that is beneficial for gut health (24).

Our stratified analysis indicated that sleep disorders and a poor-quality diet may increase the risk of constipation, with a synergistic excess risk of 27.2% (AP = 0.272, *p* = 0.002). Poor sleep and circadian rhythm disruption can lead to decreased gut microbiota diversity, impaired intestinal barrier function, and enhanced inflammatory response. These changes collectively promote visceral hypersensitivity (25, 26). Moreover, a low-fiber, high-fat, and high-sugar diet exacerbates gastrointestinal motility dysfunction (27), potentially affecting colonic motility and intestinal responsiveness.

For fecal incontinence, when the HEI-2020 score was modeled as a continuous variable, it exhibited a negative correlation with the risk of fecal incontinence (weighted adjusted OR: 0.86, 95% CI: 0.79–0.95, *p* = 0.004). However, this protective effect was attenuated after survey weighting, particularly among individuals in the highest quartile compared to those in the lowest quartile (OR: 0.77, 95% CI: 0.58–1.02, *p* = 0.066). This association is further corroborated by Zhang et al.’s cross-sectional analysis of the NHANES 2005–2010 data (15). This analysis demonstrated that individuals with optimal HEI-2015 scores (≥70) had a 31% lower odds of fecal incontinence compared to those with inadequate dietary quality (OR: 0.69, 95% CI: 0.52–0.91, *p* = 0.011). This underscores the robustness of the relationship between dietary quality and fecal incontinence.

The WQS regression model identified fatty acid ratio (a moderation component) as the primary contributor, accounting for 35.2% of the weight. Sodium intake (a moderation component) was the second most influential factor, contributing 26.0% of the weight. Similarly, when investigating the association between the individual dietary components of the HEI-2020 score and fecal incontinence using multivariate logistic regression analysis, no significant association was observed between sodium intake and fecal incontinence risk (Supplementary Table 6).

The identification of fatty acid ratio as the primary contributing component in the WQS regression model suggests a potential mechanistic link between dietary lipid balance and fecal incontinence pathophysiology. Emerging evidence indicates that fatty acid composition may influence gastrointestinal function through multiple pathways. First, the ratio of omega-3 to omega-6 polyunsaturated fatty acids modulates inflammatory processes through eicosanoid production and prostaglandin signaling pathways (28–30). Chronic, low-grade inflammation of the colonic mucosa is associated with altered rectal compliance and hypersensitivity (31, 32). These could exacerbate fecal urgency. Second, experimental studies indicate that specific fatty acid profiles can influence intestinal barrier integrity by modulating tight junction proteins (33–35). Among these, polyunsaturated and monounsaturated fatty acids are beneficial for maintaining intestinal mucosal integrity, reducing inflammation, and improving the microecological balance. In contrast, saturated fatty acids may compromise the intestinal barrier and promote inflammation (36, 37). This potentially alters the stool consistency and rectal reservoir capacity.

The lack of a significant association between the HEI-2020 and diarrhea aligns with the multifactorial etiology of diarrheal episodes. These are more likely driven by acute infections, bile acid malabsorption, or secretory pathologies than by chronic dietary patterns (38, 39).

The present study capitalized on the sampling framework of the NHANES and employed logistic regression analysis to examine the association between HEI-2020 scores and bowel habits. Additionally, WQS regression was utilized to estimate the mixture effects and weights of the 13 components constituting the HEI-2020 score. To enhance the reliability of our findings, we used a large sample size and accounted for potential confounding variables. However, this study has several limitations. First, its cross-sectional design precludes causal inferences. For example, individuals with constipation may selectively consume high-fiber foods to alleviate their symptoms, which may have biased the observed associations. In addition, the assessment of bowel habits relied primarily on the Bristol Stool Scale and standardized questionnaires, without the involvement of gastroenterologists, to confirm diagnoses or provide additional details to clarify the classification of fecal incontinence, diarrhea, and constipation. This study used a sample from the United States, and further research is needed to validate whether our findings can be generalized to other demographic populations. Unmeasured confounders, such as variations in the gut microbiota composition, may have influenced the observed associations. Future prospective cohort studies should be considered to elucidate the temporal relationship between dietary quality and bowel symptoms. The incorporation of objective biomarkers such as fecal short-chain fatty acids and microbiota profiling could help clarify the causal pathways. Additionally, mechanistic experiments such as fecal microbiota transplantation in diet-controlled models may provide deeper insights into the biological mechanisms underlying these associations.

## 5 Conclusion

Higher HEI-2020 scores were associated with a reduced risk of fecal incontinence and constipation. Adherence to the HEI-2020 guidelines may enhance gastrointestinal health by mitigating abnormalities in bowel habits.

## Data Availability

The original contributions presented in the study are included in the article/Supplementary material, further inquiries can be directed to the corresponding author.
