# Supplementary material for: Healthy Eating Index-2020 and bowel habits: a cross-sectional analysis of NHANES
Source: Front Nutr. 2025 Jun 13;12:1578124. doi: 10.3389/fnut.2025.1578124 (PMC12202417; doi:10.3389/fnut.2025.1578124)
Supplement: Supplementary file 1 [file Table_1.docx]

Supplementary Material

# Supplementary Figures and Tables

## Supplementary Figures

**Supplementary Figure 1.** Contribution weights of dietary components in the WQS models.


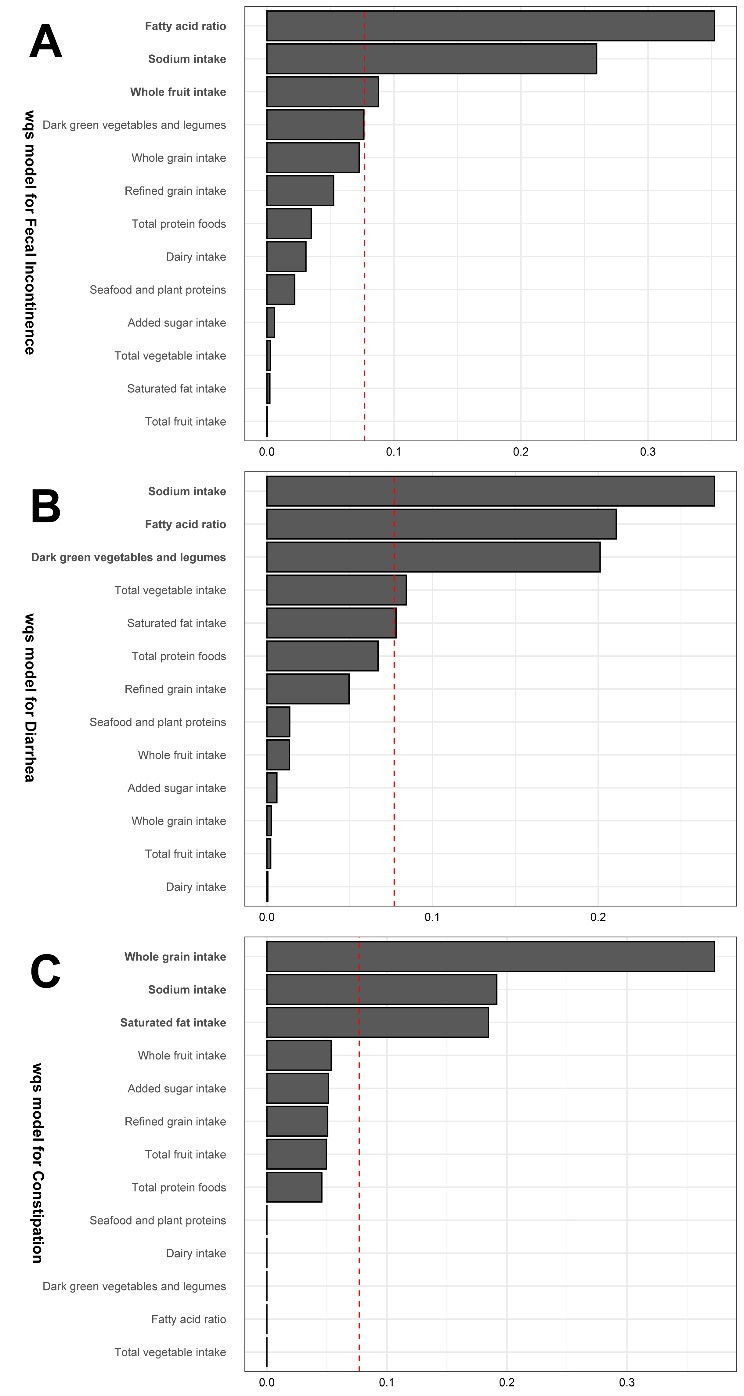


Contribution weights of 13 dietary components in the WQS regression models for bowel habits. Panels show the following models: **(A)** Fecal Incontinence Model. **(B)** Diarrhea Model. **(C)** Constipation Model. All WQS models were adjusted for age, sex, education level, family income, body mass index, alcohol use, physical activity, diabetes, hypertension, and sleep disorders.

## Supplementary Tables

**Supplementary Table 1.** Univariate logistic regression analysis of the association between HEI-2020 scores and bowel habits.

|  | Fecal Incontinence | | Diarrhea | | Constipation | |
| --- | --- | --- | --- | --- | --- | --- |
| Variable | OR (95% CI) | P | OR (95% CI) | P | OR (95% CI) | P |
| HEI-2020 scores | 1.00 (0.99~1.01) | 0.957 | 1.00 (0.99~1.00) | 0.165 | 0.98 (0.98~0.99) | <0.001 |
| Sex |  |  |  |  |  |  |
| Male | 1(Ref) |  | 1(Ref) |  |  |  |
| Female | 1.22 (1.07~1.38) | 0.003 | 1.29 (1.12~1.48) | <0.001 | 2.84 (2.48~3.25) | <0.001 |
| Age | 1.03 (1.03~1.03) | <0.001 | 1.01 (1.01~1.02) | <0.001 | 0.99 (0.99~0.99) | <0.001 |
| Race |  |  |  |  |  |  |
| Mexican American | 1(Ref) |  | 1(Ref) |  |  |  |
| Non-Hispanic | 1.07 (0.78~1.45) | 0.673 | 1.02 (0.77~1.36) | 0.874 | 1.28 (1.00~1.65) | 0.050 |
| White | 1.57 (1.29~1.91) | <0.001 | 0.77 (0.64~0.93) | 0.006 | 0.94 (0.79~1.12) | 0.510 |
| Black | 1.29 (1.03~1.63) | 0.027 | 0.96 (0.77~1.19) | 0.706 | 1.50 (1.24~1.82) | <0.001 |
| Other | 1.29 (0.88~1.87) | 0.187 | 0.88 (0.60~1.29) | 0.510 | 0.88 (0.61~1.26) | 0.483 |
| Education |  |  |  |  |  |  |
| Less than high school | 1(Ref) |  | 1(Ref) |  |  |  |
| High school or higher | 0.78 (0.68~0.90) | <0.001 | 0.57 (0.49~0.66) | <0.001 | 0.80 (0.7~0.91) | 0.001 |
| Family Income |  |  |  |  |  |  |
| Low | 1(Ref) |  | 1(Ref) |  |  |  |
| Medium | 1 .00(0.86~1.16) | 0.997 | 0.71 (0.60~0.83) | <0.001 | 0.77 (0.67~0.89) | <0.001 |
| High | 0.73 (0.62~0.87) | <0.001 | 0.58 (0.48~0.69) | <0.001 | 0.52 (0.45~0.61) | <0.001 |
| Body Mass Index | 1.02 (1.02~1.03) | <0.001 | 1.03 (1.02~1.04) | <0.001 | 0.98 (0.97~0.99) | <0.001 |
| Alcohol Use |  |  |  |  |  |  |
| No | 1(Ref) |  | 1(Ref) |  |  |  |
| Yes | 0.87 (0.76~1.00) | 0.047 | 0.83 (0.71~0.96) | 0.012 | 0.61 (0.54~0.69) | <0.001 |
| Physical Activity |  |  |  |  |  |  |
| Low | 1(Ref) |  | 1(Ref) |  |  |  |
| Moderate | 0.63 (0.55~0.73) | <0.001 | 0.71 (0.61~0.83) | <0.001 | 0.84 (0.74~0.96) | 0.008 |
| High | 0.44 (0.34~0.57) | <0.001 | 0.59 (0.45~0.76) | <0.001 | 0.69 (0.56~0.86) | 0.001 |
| Diabetes |  |  |  |  |  |  |
| No | 1(Ref) |  | 1(Ref) |  |  |  |
| Yes | 2.05 (1.8~2.33) | <0.001 | 1.52 (1.32~1.75) | <0.001 | 0.78 (0.69~0.88) | <0.001 |
| Hypertension |  |  |  |  |  |  |
| No | 1(Ref) |  | 1(Ref) |  |  |  |
| Yes | 2.06 (1.77~2.41) | <0.001 | 1.57 (1.31~1.88) | <0.001 | 0.87 (0.73~1.05) | 0.147 |
| Sleep Disorders |  |  |  |  |  |  |
| No | 1(Ref) |  | 1(Ref) |  |  |  |
| Yes | 1.93 (1.69~2.21) | <0.001 | 1.55 (1.33~1.8) | <0.001 | 1.15 (1.00~1.32) | 0.047 |

**Supplementary Table 2.** Multivariate logistic regression analysis of the association between HEI-2020 scores and bowel habits in subgroups.

| Subgroup | N | Adjusted  OR (95% CI) | P | P for interaction |
| --- | --- | --- | --- | --- |
| **Fecal Incontinence** |  |  |  |  |
| Stratified by Sex |  |  |  |  |
| Male | 5579 | 0.93 (0.84~1.03) | 0.185 | 0.703 |
| Female | 6011 | 0.87 (0.8~0.95) | 0.003 |  |
| Stratified by Age |  |  |  |  |
| < 65 | 8833 | 0.88 (0.81~0.96) | 0.005 | 0.293 |
| ≥ 65 | 2757 | 0.91 (0.81~1.01) | 0.089 |  |
| Stratified by Physical Activity |  |  |  |  |
| Low | 6137 | 0.89 (0.82~0.97) | 0.007 | 0.765 |
| Moderate | 4107 | 0.92 (0.81~1.04) | 0.170 |  |
| High | 1346 | 0.90 (0.68~1.18) | 0.430 |  |
| Stratified by Diabetes |  |  |  |  |
| No | 10016 | 0.88 (0.80~0.97) | 0.014 | 0.976 |
| Yes | 1574 | 0.91 (0.83~1.00) | 0.043 |  |
| Stratified by Hypertension |  |  |  |  |
| No | 7033 | 0.89 (0.83~0.96) | 0.004 | 0.994 |
| Yes | 4557 | 0.92 (0.79~1.07) | 0.268 |  |
| Stratified by Sleep Disorders |  |  |  |  |
| No | 8814 | 0.93 (0.86~1.01) | 0.093 | 0.138 |
| Yes | 2776 | 0.84 (0.74~0.94) | 0.003 |  |
| **Diarrhea** |  |  |  |  |
| Stratified by Sex |  |  |  |  |
| Male | 5579 | 1.01 (0.90~1.13) | 0.871 | 0.095 |
| Female | 6011 | 0.90 (0.81~0.99) | 0.034 |  |
| Stratified by Age |  |  |  |  |
| < 65 | 8833 | 0.94 (0.86~1.02) | 0.152 | 0.841 |
| ≥ 65 | 2757 | 0.96 (0.84~1.10) | 0.567 |  |
| Stratified by Physical Activity |  |  |  |  |
| Low | 6137 | 0.89 (0.81~0.98) | 0.019 | 0.123 |
| Moderate | 4107 | 1.07 (0.93~1.23) | 0.332 |  |
| High | 1346 | 0.98 (0.74~1.28) | 0.855 |  |
| Stratified by Diabetes |  |  |  |  |
| No | 10016 | 0.93 (0.84~1.03) | 0.185 | 0.640 |
| Yes | 1574 | 0.95 (0.86~1.06) | 0.395 |  |
| Stratified by Hypertension |  |  |  |  |
| No | 7033 | 0.93 (0.86~1.01) | 0.093 | 0.572 |
| Yes | 4557 | 1.01 (0.84~1.21) | 0.935 |  |
| Stratified by Sleep Disorders |  |  |  |  |
| No | 8814 | 0.92 (0.84~1.01) | 0.075 | 0.654 |
| Yes | 2776 | 1.00 (0.87~1.14) | 0.962 |  |
| **Constipation** |  |  |  |  |
| Stratified by Sex |  |  |  |  |
| Male | 5579 | 0.790 (0.69~0.90) | <0.001 | 0.735 |
| Female | 6011 | 0.81 (0.75~0.87) | <0.001 |  |
| Stratified by Age |  |  |  |  |
| < 65 | 8833 | 0.81 (0.75~0.87) | <0.001 | 0.819 |
| ≥ 65 | 2757 | 0.79 (0.68~0.90) | 0.001 |  |
| Stratified by Physical Activity |  |  |  |  |
| Low | 6137 | 0.77 (0.70~0.84) | <0.001 | 0.152 |
| Moderate | 4107 | 0.87 (0.78~0.98) | 0.020 |  |
| High | 1346 | 0.79 (0.63~1.00) | 0.049 |  |
| Stratified by Diabetes |  |  |  |  |
| No | 10016 | 0.81 (0.75~0.88) | <0.001 | 0.434 |
| Yes | 1574 | 0.78 (0.70~0.87) | <0.001 |  |
| Stratified by Hypertension |  |  |  |  |
| No | 7033 | 0.78 (0.73~0.84) | <0.001 | 0.086 |
| Yes | 4557 | 0.93 (0.77~1.12) | 0.454 |  |
| Stratified by Sleep Disorders |  |  |  |  |
| No | 8814 | 0.84 (0.78~0.90) | <0.001 | **0.030** |
| Yes | 2776 | 0.71 (0.62~0.81) | <0.001 |  |

The model adjusted for age, sex, education level, family income, body mass index, alcohol use, physical activity, diabetes, hypertension, and sleep disorders.

**Supplementary Table 3.** Interaction analysis of sleep disorders and HEI-2020 scores in constipation risk.

| Measure | Method | Estimates | Low 95%CI | High 95%CI | P |
| --- | --- | --- | --- | --- | --- |
| Without sleep disorders and high HEI-2020 scores |  | 1(ref) |  |  |  |
| With sleep disorders and high HEI-2020 scores |  | OR: 0.925 | 0.737 | 1.161 | 0.502 |
| Without sleep disorders and low HEI-2020 scores |  | OR: 1.336 | 1.154 | 1.546 | <0.001 |
| With sleep disorders and low HEI-2020 scores |  | OR: 1.733 | 1.437 | 2.089 | <0.001 |
|  | Multiplicative scale | 1.402 | 1.051 | 1.869 | 0.021 |
|  | RERI | 0.471 | 0.121 | 0.821 | 0.004 |
|  | AP | 0.272 | 0.091 | 0.453 | 0.002 |
|  | SI | 2.805 | 0.893 | 8.811 | <0.001 |

HEI-2020 scores were divided into high HEI-2020 scores group and low HEI-2020 scores group by median. The model adjusted for age, sex, education level, family income, body mass index, alcohol use, physical activity, diabetes, and hypertension.

**Supplementary Table 4.** WQS model analysis of the relationship between the mixed effects of the 13 dietary components of HEI-2020 scores and bowel habits.

| Bowel Habits | OR (95%CI) | P value |
| --- | --- | --- |
| Fecal Incontinence | 0.83 (0.72~0.97) | 0.017 |
| Diarrhea | 1.01 (0.85~1.20) | 0.951 |
| Constipation | 0.82 (0.70~0.95) | 0.008 |

The WQS model adjusted for age, sex, education level, family income, body mass index, alcohol use, physical activity, diabetes, hypertension, and sleep disorders.

**Supplementary Table 5.** WQS model analysis of the relationship between the 13 dietary components of HEI-2020 scores and bowel habits

| Exposed variable | Description | Weight |
| --- | --- | --- |
| **Fecal Incontinence** |  |  |
| HEI2020_FATTYACID | Fatty acid ratio | 0.3524 |
| HEI2020_SODIUM | Sodium intake | 0.2595 |
| HEI2020_FRT | Whole fruit intake | 0.0878 |
| HEI2020_GREENNBEAN | Dark green vegetables and legumes | 0.0763 |
| HEI2020_WHOLEGRAIN | Whole grain intake | 0.0726 |
| HEI2020_REFINEDGRAIN | Refined grain intake | 0.0525 |
| HEI2020_TOTALPRO | Total protein foods | 0.0350 |
| HEI2020_DAIRY | Dairy intake | 0.0307 |
| HEI2020_SEAPLANTPRO | Seafood and plant proteins | 0.0218 |
| HEI2020_ADDEDSUGAR | Added sugar intake | 0.0059 |
| HEI2020_VEG | Total vegetable intake | 0.0030 |
| HEI2020_SATFAT | Saturated fat intake | 0.0023 |
| HEI2020_TOTALFRT | Total fruit intake | 0.0001 |
| **Diarrhea** |  |  |
| HEI2020_SODIUM | Sodium intake | 0.2702 |
| HEI2020_FATTYACID | Fatty acid ratio | 0.2109 |
| HEI2020_GREENNBEAN | Dark green vegetables and legumes | 0.2012 |
| HEI2020_VEG | Total vegetable intake | 0.0842 |
| HEI2020_SATFAT | Saturated fat intake | 0.0780 |
| HEI2020_TOTALPRO | Total protein foods | 0.0671 |
| HEI2020_REFINEDGRAIN | Refined grain intake | 0.0496 |
| HEI2020_SEAPLANTPRO | Seafood and plant proteins | 0.0138 |
| HEI2020_FRT | Whole fruit intake | 0.0136 |
| HEI2020_ADDEDSUGAR | Added sugar intake | 0.0060 |
| HEI2020_WHOLEGRAIN | Whole grain intake | 0.0027 |
| HEI2020_TOTALFRT | Total fruit intake | 0.0023 |
| HEI2020_DAIRY | Dairy intake | 0.0005 |
| **Constipation** |  |  |
| HEI2020_WHOLEGRAIN | Whole grain intake | 0.3729 |
| HEI2020_SODIUM | Sodium intake | 0.1915 |
| HEI2020_SATFAT | Saturated fat intake | 0.1846 |
| HEI2020_FRT | Whole fruit intake | 0.0536 |
| HEI2020_ADDEDSUGAR | Added sugar intake | 0.0514 |
| HEI2020_REFINEDGRAIN | Refined grain intake | 0.0506 |
| HEI2020_TOTALFRT | Total fruit intake | 0.0497 |
| HEI2020_TOTALPRO | Total protein foods | 0.0457 |
| HEI2020_SEAPLANTPRO | Seafood and plant proteins | 0.0000 |
| HEI2020_DAIRY | Dairy intake | 0.0000 |
| HEI2020_GREENNBEAN | Dark green vegetables and legumes | 0.0000 |
| HEI2020_FATTYACID | Fatty acid ratio | 0.0000 |
| HEI2020_VEG | Total vegetable intake | 0.0000 |

The WQS model adjusted for age, sex, education level, family income, body mass index, alcohol use, physical activity, diabetes, hypertension, and sleep disorders.

**Supplementary Table 6.** Multivariate logistic regression analysis of the association between HEI-2015 components and bowel habits.

| Exposed variable | Description | Adjusted  OR (95% CI) | P |
| --- | --- | --- | --- |
| **Fecal Incontinence** |  |  |  |
| HEI2020_TOTALFRT | Total fruit intake | 0.95 (0.92~0.99) | **0.010** |
| HEI2020_FRT | Whole fruit intake | 0.98 (0.94~1.01) | 0.237 |
| HEI2020_VEG | Total vegetable intake | 0.93 (0.89~0.98) | **0.008** |
| HEI2020_GREENNBEAN | Dark green vegetables and legumes | 0.98 (0.94~1.02) | 0.410 |
| HEI2020_TOTALPRO | Total protein foods | 0.93 (0.87~1.00) | **0.045** |
| HEI2020_SEAPLANTPRO | Seafood and plant proteins | 0.98 (0.95~1.02) | 0.317 |
| HEI2020_WHOLEGRAIN | Whole grain intake | 0.99 (0.97~1.02) | 0.569 |
| HEI2020_DAIRY | Dairy intake | 0.99 (0.97~1.02) | 0.522 |
| HEI2020_FATTYACID | Fatty acid ratio | 0.97 (0.95~1.00) | **0.023** |
| HEI2020_REFINEDGRAIN | Refined grain intake | 1.01 (0.99~1.03) | 0.425 |
| HEI2020_SODIUM | Sodium intake | 1.01 (0.99~1.03) | 0.382 |
| HEI2020_ADDEDSUGAR | Added sugar intake | 0.98 (0.95~1.00) | **0.040** |
| HEI2020_SATFAT | Saturated fat intake | 0.96 (0.94~0.98) | **0.001** |
| **Diarrhea** |  |  |  |
| HEI2020_TOTALFRT | Total fruit intake | 0.96 (0.92~1.00) | 0.032 |
| HEI2020_FRT | Whole fruit intake | 0.95 (0.92~0.99) | **0.014** |
| HEI2020_VEG | Total vegetable intake | 1.00 (0.95~1.06) | 0.885 |
| HEI2020_GREENNBEAN | Dark green vegetables and legumes | 1.02 (0.98~1.07) | 0.353 |
| HEI2020_TOTALPRO | Total protein foods | 1.05 (0.98~1.14) | 0.172 |
| HEI2020_SEAPLANTPRO | Seafood and plant proteins | 0.99 (0.95~1.03) | 0.632 |
| HEI2020_WHOLEGRAIN | Whole grain intake | 0.97 (0.94~0.99) | **0.017** |
| HEI2020_DAIRY | Dairy intake | 0.95 (0.93~0.98) | **<0.001** |
| HEI2020_FATTYACID | Fatty acid ratio | 1.02 (1.00~1.05) | 0.060 |
| HEI2020_REFINEDGRAIN | Refined grain intake | 0.99 (0.97~1.01) | 0.432 |
| HEI2020_SODIUM | Sodium intake | 1.00 (0.97~1.02) | 0.923 |
| HEI2020_ADDEDSUGAR | Added sugar intake | 0.98 (0.96~1.01) | 0.180 |
| HEI2020_SATFAT | Saturated fat intake | 1.03 (1.01~1.06) | **0.015** |
| **Constipation** |  |  |  |
| HEI2020_TOTALFRT | Total fruit intake | 0.94 (0.90~0.97) | **<0.001** |
| HEI2020_FRT | Whole fruit intake | 0.93 (0.90~0.97) | **<0.001** |
| HEI2020_VEG | Total vegetable intake | 0.88 (0.84~0.93) | **<0.001** |
| HEI2020_GREENNBEAN | Dark green vegetables and legumes | 0.92 (0.88~0.95) | **<0.001** |
| HEI2020_TOTALPRO | Total protein foods | 0.9 (0.84~0.95) | **<0.001** |
| HEI2020_SEAPLANTPRO | Seafood and plant proteins | 0.92 (0.88~0.95) | **<0.001** |
| HEI2020_WHOLEGRAIN | Whole grain intake | 0.96 (0.93~0.98) | **<0.001** |
| HEI2020_DAIRY | Dairy intake | 0.99 (0.97~1.01) | 0.302 |
| HEI2020_FATTYACID | Fatty acid ratio | 0.98 (0.96~1.00) | **0.038** |
| HEI2020_REFINEDGRAIN | Refined grain intake | 1.00 (0.98~1.02) | 0.807 |
| HEI2020_SODIUM | Sodium intake | 1.02 (1.00~1.04) | 0.073 |
| HEI2020_ADDEDSUGAR | Added sugar intake | 0.94 (0.92~0.96) | **<0.001** |
| HEI2020_SATFAT | Saturated fat intake | 0.99 (0.97~1.02) | 0.512 |

The model adjusted for age, sex, education level, family income, body mass index, alcohol use, physical activity, diabetes, hypertension, and sleep disorders.
